# Detached Twig Assay to Evaluate Bacterial Canker on Peaches

**DOI:** 10.3390/mps9020034

**Published:** 2026-02-28

**Authors:** Bilgehan A. Geylani, Stephen M. Parris, Jhulia Gelain, Guido Schnabel, Ksenija Gasic

**Affiliations:** Plant and Environmental Sciences Department, Clemson University, Clemson, SC 29634, USA; bgeylan@clemson.edu (B.A.G.); sparri2@clemson.edu (S.M.P.); jgelain@clemson.edu (J.G.); schnabe@clemson.edu (G.S.)

**Keywords:** *Pseudomonas syringae* pv. *syringae*, lab assay, ImageJ, phenotyping, disease response, bacterial canker

## Abstract

*Pseudomonas syringae* pv. *syringae* (*Pss*) is the causal agent of bacterial canker, a disease that can result in yield losses, aerial tissue damage, and tree mortality in stone fruits worldwide. Peach, one of the major stone fruit crops, experiences significant yield losses and tree mortality attributed to bacterial canker in the United States. As the second-largest peach-producing state, South Carolina faces direct and significant impacts due to *Pss*. Early evaluations of peach scion responses to *Pss* infection have relied primarily on circumstantial field observations in rootstock trials. Although laboratory evaluations in peach have been reported, these studies primarily focused on pathogen virulence testing or small accession sets and did not establish a standardized, scalable detached twig protocol for systematic germplasm phenotyping. The absence of a clearly described laboratory assay has limited reproducible and large-scale evaluation of bacterial canker tolerance in peach. To address this gap, a detached dormant twig assay, previously developed for cherry, was adapted and optimized for peach. Dormant shoots from nine peach accessions were cut into 10 cm segments, surface-sterilized, and inoculated with a *Pss* suspension prepared in 10 mM MgCl_2_ buffer or with the buffer alone. After six weeks of incubation, inner bark lesion size was evaluated visually and quantified using ImageJ. A newly developed visual rating scale was established and compared with quantitative lesion measurements. Spearman correlation analysis showed strong positive correlations between visual disease scores and ImageJ-based lesion measurements across two independent replicates (ρ = 0.80–1.00, *p* < 0.01), while shoot segment diameter showed weak-to-moderate negative correlations with disease severity. This adapted and consolidated dormant twig assay provides a practical, reproducible, and scalable method for phenotyping bacterial canker tolerance in peach and supports future germplasm screening and breeding efforts.

## 1. Introduction

Bacterial canker, caused by *Pseudomonas syringae* pv. *syringae* van Hall (*Pss*), is one of the most significant threats to stone fruit production (*Prunus* spp.) worldwide and is a major cause of peach (*P. persica* [L.] Batsch) orchard decline in the Southeast U.S. [1,2]. Peach is an important stone fruit crop in the United States, with a total annual production (fresh market and processing fruit) of 747,811 tons reported in 2024/2025 [3]. California is the leading producer of both processing and fresh-market peaches followed by South Carolina, which ranks second with 91,600 tons of fresh-market production valued at an estimated $188.7 million, based on USDA-reported unit prices for 2024/2025 [4]. *Pss* is generally considered a weak or opportunistic pathogen that requires favorable environmental conditions to infect plants [5]. Due to the pathogen’s epiphytic nature, *Pss* overwinters and colonizes on plant parts such as dormant buds, leaves and plant debris, which serve as an inoculum source for forthcoming infections when the tree is predisposed by other factors [6,7]. Pruning, winter injuries, mechanical damage, leaf drop scars, and stomata provide an opening for *Pss* to infect a tree [8,9]. *Pss* is an ice-nucleation-active bacteria that reduces a plant’s ability to supercool and increases its sensitivity to freezing temperatures below 2 °C, making winter frosts a critical predisposing factor for disease development [10,11]. Additionally, stresses from biotic and/or abiotic factors, such as those from ring nematodes (*Criconemoides xenoplax* Raski syn. *Mesocriconema xenoplax* Raski) which feed on roots and can cause peach tree short life syndrome (PTSL), also predispose trees to *Pss* infection [12,13].

Due to the limited effectiveness of chemical bactericides and the potential for development of resistant pathogen strains, *Pss* management relies primarily on cultural practices aimed at reducing tree stress associated with cold winter injury, pathogenic nematode presence, and nutrition management [14,15,16,17,18,19]. However, these approaches usually provide inconsistent and unreliable control under variable environmental conditions [19]; thus, genetic tolerance is viewed as the most sustainable and long-term strategy for *Pss* management [2,20,21,22]. Despite its importance, tolerance to bacterial canker among peach cultivars has not been systematically characterized, in part due to a lack of standardized and reproducible phenotyping methods. Although one study reported responses of peach and other stone fruit cultivars grown under greenhouse conditions in Pakistan [23], and laboratory-based pathogenicity assays including peach have been described using vermiculite-based systems [24], these studies primarily focused on seedling response or pathogen virulence testing. A consolidated and standardized detached dormant twig protocol optimized for peach germplasm phenotyping has not been clearly established.

Comprehensive studies on *Pss* tolerance in stone fruit crops have been conducted only on sweet cherry (*Prunus avium*) [20,21,22,25]. Such studies involved evaluating field- and lab-based responses and determining the genetic basis of tolerance in cherry germplasm. Only two studies focusing on the genetic component of *Pss* tolerance in *Prunus* crops (almond [25] and apricot [26]) are available, with none conducted on peach. Previous studies on peach focused mostly on the impact of management, nutrients, nematode infestation and rootstock effect on peach susceptibility to *Pss* [13,17,18,27,28,29].

The quality of phenotyping is very important for downstream analyses, especially when identifying genes underlying specific traits, and could be hampered due to confounding factors produced by experimental conditions and human observations [30,31]. In addition, the availability of detailed protocols for both field- and lab-based disease assays is scarce and rarely result in comparable data [21,22,32,33]. *Pss* studies in cherry have successfully utilized laboratory-based leaf and detached shoot assays by performing uniform inoculation and controlled disease development [21,33]. Data collection is image-based and obtained visually via comparison to previously created rating scales and/or utilization of automated image analysis tools like ImageJ [21,22,32,33].

The lack of studies evaluating bacterial canker tolerance in peach germplasm has limited breeding efforts aimed at developing new cultivars tolerant to this devastating disease. To enable future genetic studies and facilitate breeding for tolerance to *Pss* in peach, we adapted a previously established dormant twig assay from sweet cherry for use in peach [22,33]. The following sections detail the methods used to collect dormant twigs from nine peach accessions, inoculate them with *Pss*, incubate them, and evaluate disease severity to assess tolerance. This dormant twig assay provides a practical and reliable phenotyping approach for assessing bacterial canker tolerance in peach germplasm. The resulting phenotyping data can be integrated with genomic or transcriptomic resources to identify candidate genes and genomic regions associated with tolerance to *Pss* in peach.

## 2. Experimental Design

**Figure 1:**
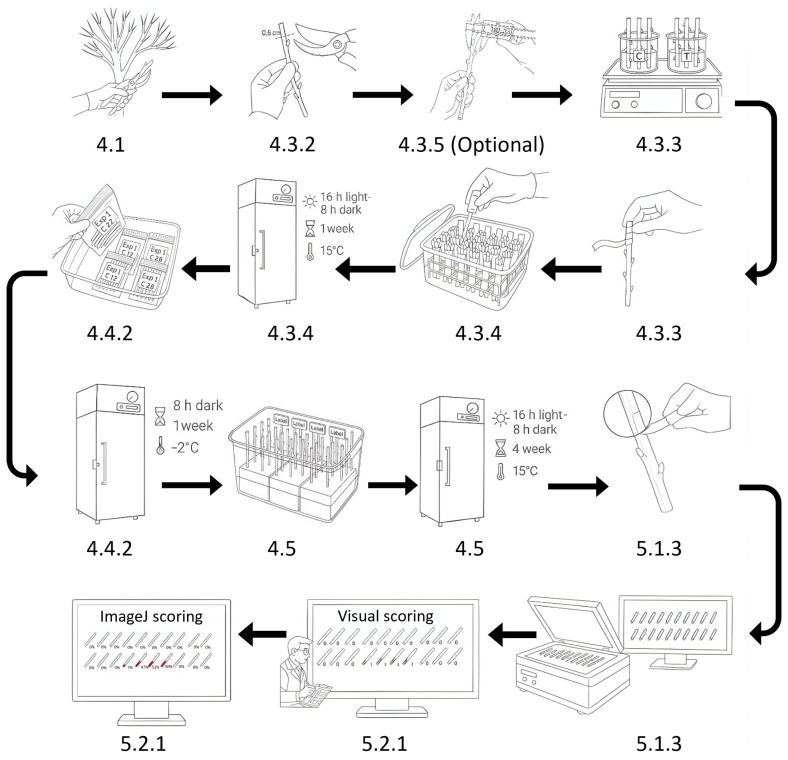
Overall workflow of the detached dormant twig assay. Arrays designate the order of steps in the protocol. Numbers correspond with the steps described in the Procedure.

The objective of this study was to adapt a phenotyping protocol previously described in sweet cherry to assess variation in tolerance to *Pss* in peach germplasm [22,33]. Dormant branches (twigs), collected from nine peach accessions, were cut into ten 10 cm long segments per accession and inoculated with *Pss* suspension (treatment), or mock inoculated with buffer (control), under controlled laboratory conditions (Figure 1). For each accession, both control and treatment groups were established, and the experiment was performed in two independent replications. The protocol described in this peer-reviewed article is published on protocol.io (DOI: 10.17504/protocols.io.3byl46n62go5/v1) and is included in the Supplementary Materials.

### 2.1. Materials

*1.*  Gloves (TouchNTuff^®^, Ansell, Iselin, NJ, USA);
*2.*  Alcohol, 70% (Thermo Fisher Scientific Inc., Waltham, MA, USA);
*3.*  Flagging tape (for bundling each cultivar’s dormant shoots after collection; each shoot bundle should be labeled with genotype name on the tape; FisherBrand, Thermo Fisher Scientific Inc., Waltham, MA, USA);
*4.*  Paper wipes (KimTech, ULINE, Pleasant Prairie, WI, USA);
*5.*  Plastic plates (disposable polystyrene weighing dishes for overnight drying, 1 per accession) (Sigma-Aldrich, St. Louis, MO, USA);
*6.*  Plastic pot labels (Hummert International, Earth City, MO, USA);
*7.*  Parafilm (Parafilm M Lab Film, ULINE, Pleasant Prairie, WI, USA);
*8.*  Floral foam bricks (OASIS^®^ Standard Floral Foam Maxlife, Kent, OH, USA);
*9.*  Ziplock bags (for second incubation) (S.C. & Son Inc. Johnson, Racine, WI, USA);
*10.*  Autoclavable bags (Thermo Fisher Scientific Inc., Waltham, MA, USA);
*11.*  Bleach (Clorox™, Oakland, CA, USA);
*12.*  Razor blades (for peeling) (Ihc World LLC, Ellicott City, MD, USA);
*13.*  Absorbent surface liners (as a bench protector during the peeling process) (Thermo Fisher Scientific Inc., Waltham, MA, USA);
*14.*  Pathogen: *Pseudomonas syringae* pv. *syringae* isolate *Pss* S2, provided by Dr. Hehe Wang from Clemson University;
*15.*  Plant material: twenty 30 cm long, one-year-old dormant shoots.

### 2.2. Equipment

*1.*  Pruners (Corona Clipper Inc., Corona, CA, USA);
*2.*  Markers (Sharpie, Newell Brands, Atlanta, GA, USA);
*3.*  Tongs for transferring shoot segments during disinfection (Oneida^®^, Oneida, NY, USA);
*4.*  Digital caliper (Mitutoyo America Corporation, Aurora, IL, USA);
*5.*  Tube racks (Thermo Fisher Scientific Inc., Waltham, MA, USA);
*6.*  Plastic containers (for holding shoot segments) (Sterilite Corporation, Townsend, MA, USA);
*7.*  Beakers, 250 mL (Pyrex™, Corning Incorporated, Corning, NY, USA);
*8.*  Orbital shaker/Advanced digital shaker (Avantor Inc., Radnor, PA, USA);
*9.*  Containers, 1 L (ULINE, Pleasant Prairie, WI, USA);
*10.*  Refrigerator, 4 °C;
*11.*  Growth chamber (15 °C, 8 h dark, 16 h light) (Percival Scientific Inc., Perry, IA, USA);
*12.*  Refrigerator, −2 °C;
*13.*  Autoclavable trays (Nalgene, Thermo Fisher Scientific Inc., Wlatham, MA, USA);
*14.*  Scanner (Epson America Inc., Los Alamitos, CA, USA);
*15.*  ImageJ software v. 1.54p (National Institutes of Health, Bethesda, MD, USA);
*16.*  R software (version 4.5.1; R Core Team, Vienna, Austria);

## 3. Procedure

**Figure 2:**
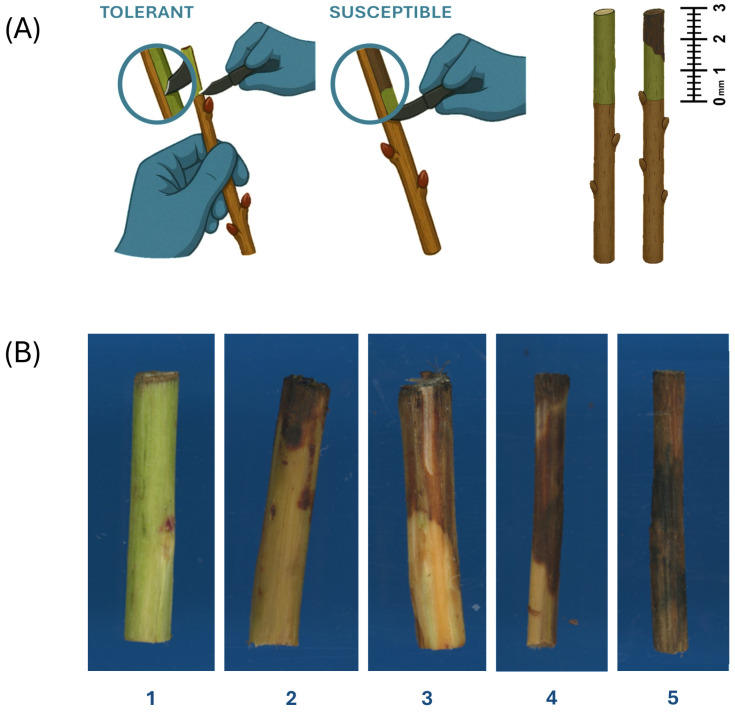
Visual scale for *Pseudomonas syringae* pv. *syringae* symptom severity evaluation based on inner bark lesion size. (**A**) Removal of the outer bark and exposure of inner bark tissue at the top 3 cm of the shoot segment; (**B**) Symptoms observed in peach with description of the scores provided in Table 1.

1. *Pseudomonas syringae* inoculum preparation
  *1.1.*  *Media Buffer Preparation*
    1.1.1. Prepare King’s B Media (KMB—recipe below) or Pseudomonas Agar F plates:
      - Add 20 g of proteose peptone, 1.5 g of K_2_HPO_4_, and 1.5 g of MgSO_4_·7H_2_O to ~900 mL of distilled water.
      - Add 10 mL of glycerol (measure accurately; viscous).
      - Add 15 mg of agar powder (BD Difco, Thermo Fisher Scientific, Waltham, MA, USA, Cat. No. DF0812-07-1).
      - Stir until fully dissolved.
      - Bring volume up to 1 L with distilled water.
      - Adjust pH to 7.2 if needed (usually not necessary).
      - Autoclave at 121 °C for 15 min.
      - Cool to ~55 °C and pour plates.
    1.1.2. Prepare Luria–Bertani (LB) broth (BD Difco, Thermo Fisher Scientific, Waltham, MA, USA Cat. No. DF0446075); pour 20 mL into each 50 mL Falcon tube.
    1.1.3. Prepare sterile 10 mM MgCl_2_ buffer:
      - The 10 mM MgCl_2_ consists of 0.952 g of anhydrous MgCl_2_ or 2.03 g of MgCl_2_ × 6 H_2_O in 1 L of DI water; autoclave.
  *1.2.*  *Revival and Plating*
    1.2.1. Streak *Pseudomonas syringae* isolate from glycerol stock preserved in Cryo media onto KMB/*Pseudomonas* agar plates.
    1.2.2. Incubate plates at 25–30 °C for 24–48 h.
  *1.3.*  *Colony Purity Check*
    1.3.1. Examine plates under 365 nm UV light.
    1.3.2. Confirm uniform fluorescence (yellow-green).
  *1.4.*  *Starter Culture Preparation*
    1.4.1. Using a sterile loop, pick a single colony from KMB.
    1.4.2. Inoculate into 20 mL of LB broth in sterile culture tubes.
    1.4.3. Incubate overnight (14–18 h) at 30 °C, 180 rpm.
  *1.5.*  *Measure Starter Culture Density*
    1.5.1. Blank spectrophotometer with LB.
    1.5.2. Measure OD_600_ of the starter culture. Typical values: 0.3–0.6.
  *1.6.*  *Log-Phase Culture Initiation*
    1.6.1. Transfer 0.2–0.4 mL of the starter culture into 20 mL of LB broth.
    1.6.2. Incubate at 30 °C, 180 rpm for ~6 h to reach the early log phase.
  *1.7.*  *Confirm Log-Phase Density*
    1.7.1. Measure OD_600_ using LB as blank.
    1.7.2. Proceed when OD_600_ ≈ 0.2–0.3 (≈2–3 × 10^8^ CFU/mL).
2. Cell Harvesting and Preparation of Inoculum
  *2.1.*  *Pellet Cells*
    2.1.1. Centrifuge cultures at 5000 rpm for 15 min.
    2.1.2. Discard supernatant.
  *2.2.*  *Wash Cells*
    2.2.1. Add 20 mL of sterile 10 mM MgCl_2_ to each pellet.
    2.2.2. Resuspend by gentle shaking.
    2.2.3. Centrifuge at 5000 rpm for 10 min.
    2.2.4. Discard supernatant.
  *2.3.*  *Final Resuspension*
    2.3.1. Add 20 mL of fresh 10 mM MgCl_2_ to the pellet.
    2.3.2. Resuspend thoroughly.
    2.3.3. Transfer the entire resuspended volume into a sterile bottle containing 580 mL of MgCl_2_ to obtain 600 mL of inoculum.
    2.3.4. Mix gently. Final concentration ≈ 1 × 10^7^ CFU/mL. Use suspension within 4 h of preparation.
3. Verification of Cell Concentration
  *3.1.*  *Serial Dilution Plating*
    3.1.1. Prepare serial 10-fold dilutions in 10 mM MgCl_2_ (10^−1^ to 10^−7^).
    3.1.2. Plate 100 µL of 10^−6^ and/or 10^−7^ dilutions onto KMB agar.
    3.1.3. Incubate at 28–30 °C overnight.
  *3.2.*  *CFU Calculation*
    3.2.1. Count colonies on plates with 30–300 CFUs.
    3.2.2. CFU/mL = (colonies × dilution factor)/volume plated (mL).
4. Detached Twig Lab Assay
  *4.1.*  *Collection of Shoots (Day 1)*
    4.1.1.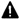 CRITICAL STEP: Collect dormant, one-year-old shoots from trees in the field.
    4.1.2. Immediately prepare shoots following directions below, or store collected shoots at 4 °C.
  *4.2.*  *Preparation of Shoots*
    4.2.1. Cut shoots into twenty 10 cm segments of uniform thickness for each cultivar.
    4.2.2. Disinfect shoot segments by immersing in bleach (0.5% sodium hypochlorite) for 5 min, stirring periodically.
    4.2.3. Rinse shoot segments in distilled water three times.
    4.2.4. Set shoot segments on benchtop to dry:
      - Prevent shoot segment overnight drying by loosely covering them with paper towels.
    4.2.5. Measure diameter of shoot segments at the tip with caliper (can be done after incubation steps):
      - **OPTIONAL STEP:** Shoot diameter can be also measured with ImageJ software on scanned images (step 5.1.5) by converting pixel-based measurement into millimeters (mm).
  *4.3.*  *Inoculation of the Shoots (Day 2)*
    4.3.1. Preparation of *Pss* inoculum:
      - Dispense actively growing *Pss* liquid cultures prepared above in steps 8–10 into 250 mL sterile beakers to a depth of approximately 1.3 cm. Use non-inoculated 10 mM MgCl_2_ solution for the mock inoculation control group.
      - Place beakers onto an orbital shaker (100 RPM) to prevent bacteria from settling.
    4.3.2. Preparation of shoot segments for inoculation: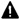 CRITICAL STEP: Trim 0.5 cm from the top of each shoot segment to remove bleach damage and expose fresh tissue.
      - Use very sharp pruners or single-edge razor blades to ensure a clean cut is obtained.
    4.3.3. Shoot inoculation:
      - For each cultivar inoculate 10 shoot segments with *Pss* (treatment) and mock-inoculate 10 shoot segments with 10 mM MgCl_2_ solution (control).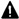 CRITICAL STEP: Submerge the freshly cut end of prepared shoot segments into the inoculum or 10 mM MgCl_2_ solution for 5 min, maintain gentle agitation (100 RPM) to ensure uniform exposure and avoid excessive shaking or frothing.
      - Remove shoot segments from beakers and wrap the inoculated end with parafilm.
    4.3.4. Preparation for co-cultivation:
      - Label separate plastic containers for treatment and control, place plastic racks in the containers and fill with tap water to 1.5 cm depth.
      - Trim 0.5 cm from the bottom of the shoot segments to expose fresh tissue.
      - Bundle 10 shoot segments labeled by sample and place in plastic racks with the freshly cut end submerged in the 1.5 cm of tap water.
      - Alternatively, shoot segments can be separated in floral foam bricks or individual containers.
      - Incubate closed containers in a growth chamber for 1 week (16 h light-8 h dark at 15 °C).
  *4.4.*  *Cold Treatment (Day 10)*
    4.4.1. After 1 week of incubation at 15 °C, remove the parafilm from the top and dry the base of shoot segments with paper tissue:
      - Sterilize the containers with bleach for later use.
    4.4.2.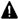 CRITICAL STEP: Place shoot segments into pre-labeled plastic zip-lock bags and transfer to a refrigerator for cold treatment at −2 °C in darkness for one week.
  *4.5.*  *Incubation (Day 17)*
    4.5.1. Prepare sterile containers for incubation by placing floral foam bricks, saturating them with tap water, and maintaining a water level of 1.5 cm throughout the experiment to ensure adequate moisture:
      - Alternatively, shoot segments could be placed in plastic racks or individual containers.
    4.5.2.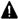 CRITICAL STEP: Incubate closed containers in a growth chamber for 4 weeks under the same conditions as described under 4.3.4.
5. *Data Collection*
  *5.1.*  *Shoot Preparation and Imaging*
    5.1.1. Remove shoot segments from the experiment and proceed with recording symptom severity:
      - If not evaluating symptom severity at the end of the experiment (too many shoot segments to process) within the same day, place shoot segments in zip-lock bags and store at 4 °C to suppress bacterial growth.
    5.1.2. First, collect an image of shoot segments for each sample using a high-resolution scanner or camera:
      - Arrange the shoot segments on the scanner tray with the control at the top and the treatment at the bottom of the tray, with the inoculated end oriented in the same direction.
      - Scan the shoot sections from both sides.
    5.1.3. Second, expose inner bark tissue to evaluate the symptom severity: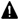 CRITICAL STEP: Remove the outer bark with surgical scalpel or single-edge razor blade from the top 3 cm of each shoot segment (Figure 2A).
    5.1.4. Trim and collect the top 3 cm of each shoot segment and discard the unpeeled base.
    5.1.5. Place peeled shoot segments onto the scanner tray and collect images.
  *5.2.*  *Evaluation of Symptom Severity*
    5.2.1. Visually evaluate symptom severity, using photographed shoot segments, on a scale from 1 to 5, where 1 represents a sample without necrotic lesion on the inner bark, and 5 represents a sample with inner bark completely covered with a necrotic lesion (Figure 2B).
    5.2.2. Quantitatively evaluate symptom severity, using photographed shoot segments, by measuring the percentage of each shoot segment’s surface area covered by necrotic lesion with ImageJ software.
  *5.3.*  *Statistical Analysis*
    Statistical analyses and data visualizations were performed in R (version 4.5.1) using the *psych* and *tidyverse* packages. Spearman’s correlation coefficients between visual and ImageJ-based scoring methods, replications, and twig diameter measurements were evaluated using the *pair.panels* function. The distribution pattern of lesion scores across replications and scoring methods was illustrated with a violin plot by using the *geom_violin* function.

**Table 1:** Description of the visual and quantitative disease severity scale developed for evaluating *Pseudomonas syringae* pv. *syringae* symptoms in peach detached dormant shoot segments manually and using ImageJ software, respectively.

Manual Score	ImageJ Score	Symptom Severity
1	0–20%	No visible symptoms or discoloration
2	21–40%	Slight lesion development
3	41–60%	Moderate lesion development
4	61–80%	Extensive lesion development
5	81–100%	Severe lesion development

## 4. Results

The adapted dormant twig assay was validated using nine peach accessions. Symptoms observed in these nine accessions were used to develop a five-point visual rating scale (Figure 2B) and were aligned with corresponding ImageJ-based lesion area percentages (Table 1). Visual scores ranged from 1 (no visible symptoms) to 5 (severe lesion development), corresponding to 0–100% lesion area.

**Figure 3:**
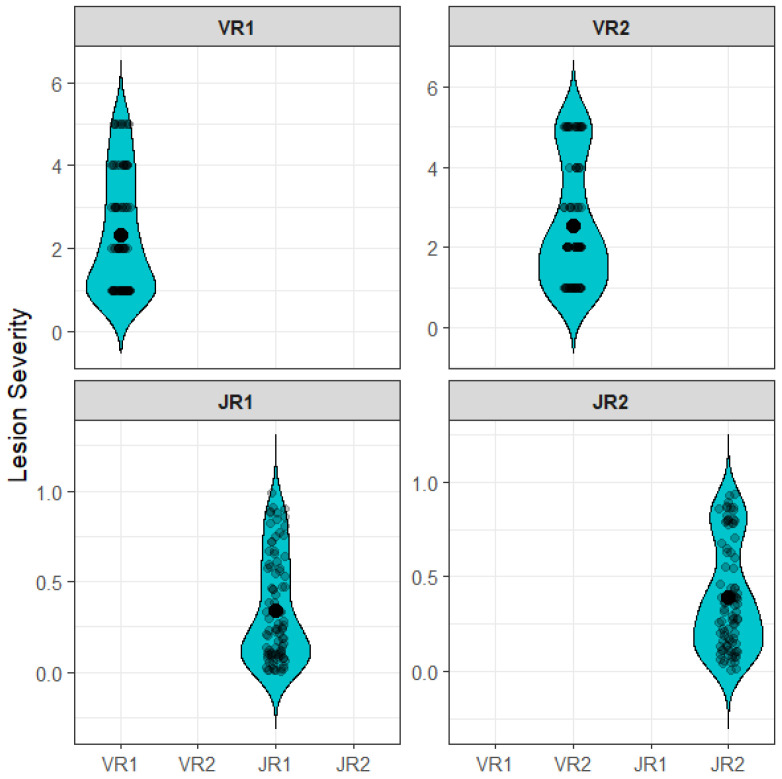
Distribution of disease severity observed in the inner bark of detached dormant twigs from nine peach accessions inoculated with *Pseudomonas syringae* pv. *syringae* (*Pss*). Visual (V) and ImageJ-based (J) measurements are shown for two replicates (R). Data presents ten observations per accession per replicate.

The assay successfully differentiated disease severity responses among the nine peach accessions (Figure 3, Table 2). Mean visual disease severity scores, based on ten twig segments per accession, ranged from 1.1 to 5.0, with overall average values of 2.32 and 2.55 in R1 and R2, respectively. ImageJ-based lesion measurements ranged from 0.08 to 0.85 in R1 with average values of 0.34 and 0.39 in R1 and R2, respectively. Dormant twig segment diameters ranged from 0.38 to 0.59 mm in R1, and mean diameters were similar between replicates (0.49 mm in R1 and 0.43 mm in R2).

**Figure 4:**
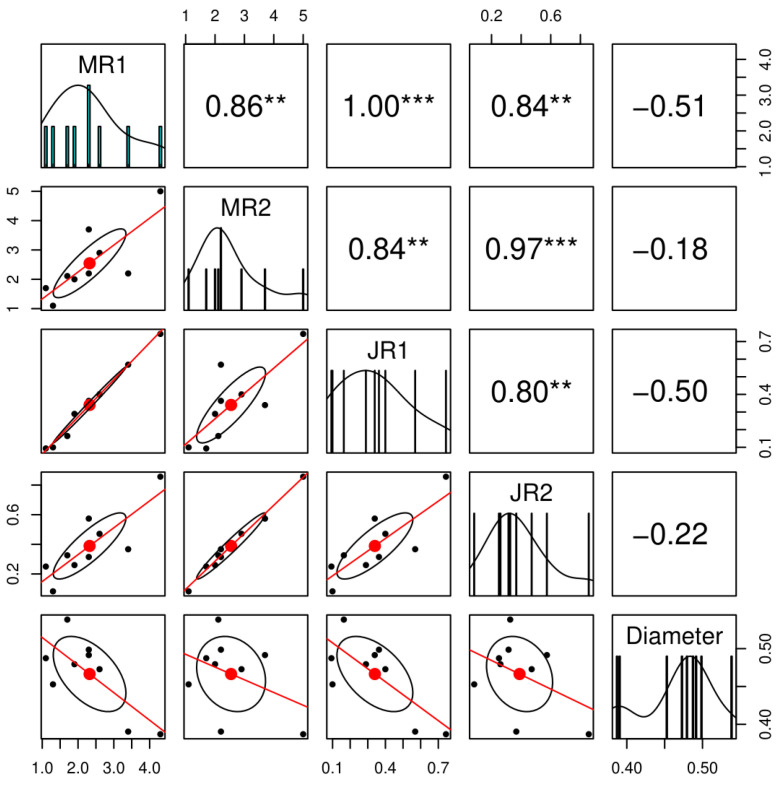
Spearman correlation matrix showing strong positive correlations between replication-specific visual (V) and ImageJ-based (J) inner bark lesion measurements, and weak negative correlations with twig segment diameter across replicates (R) in nine peach accessions inoculated with *Pseudomonas syringae* pv. *syringae* (*Pss*). Data presents ten observations per accession per replicate. Asterisks indicate significance levels for Spearman’s rank correlation coefficient (*p* < 0.01 and *p* < 0.001).

Spearman correlation analysis revealed strong and significant positive correlations between visual (VR1 and VR2) and ImageJ-based (JR1 and JR2) lesion measurements across replicates (R) (ρ = 0.80–1.00, *p* < 0.01), demonstrating high reproducibility of the assay and strong agreement between the manual and digital phenotyping approaches (Figure 4). In contrast, twig segment diameter showed weak negative correlations with disease severity, suggesting that variation in twig size did not substantially influence lesion assessment.

## 5. Discussion

Reliable and reproducible phenotyping is essential for identifying genetic variation and supporting breeding efforts in perennial crops [34]. Bacterial canker caused by *Pseudomonas syringae* pv. *syringae* is an important disease of stone fruit worldwide [1,7], and the development of genetically tolerant cultivars remains the most sustainable long-term management strategy [2,20,21,22]. However, evaluation of bacterial canker response under field conditions is challenging due to environmental variability and the opportunistic and often asymptomatic nature of the pathogen, which limits accurate assessment of disease response [8,34]. Laboratory-based detached leaf and shoot assays have been developed to enable controlled and uniform evaluation of disease development in woody crops [20,21,22,25,26]. Laboratory-based pathogenicity testing in peach has also been reported using vermiculite-based inoculation systems [24] and greenhouse seedling evaluations [23]. However, these studies primarily assessed pathogen virulence or cultivar response under different experimental frameworks and did not provide a detailed and standardized detached dormant twig protocol tailored for reproducible germplasm phenotyping in peach. In sweet cherry, detached twig assays were shown to produce symptom patterns comparable to field evaluations, whereas detached leaf assays were less representative of whole-plant responses [1]. Although such assays have been successfully applied in sweet cherry to evaluate bacterial canker responses [20,21,22], procedural details are not always fully described, and a standardized protocol for peach has not been established. Therefore, we adapted the dormant twig assay used for bacterial canker evaluation in cherry [21] and prepared a consolidated protocol to enable reproducible evaluation of bacterial canker tolerance in peach germplasm.

When applied to peach, the adapted dormant twig assay produced clear and measurable symptoms six weeks after inoculation, which is similar to cherry [21]. A five-point visual rating scale was developed based on the range and progression of symptoms observed in this study. Alignment of visual scores with ImageJ-derived lesion area measurements provided a standardized framework for both qualitative and quantitative assessment. The strong agreement between visual and image-based measurements across replicates confirms that the visual scale is suitable for rapid screening while maintaining consistency with quantitative image analysis. Consistent lesion severity patterns across replicates further demonstrate the reproducibility of the adapted assay. Although a weak negative association between twig diameter and lesion severity was detected, regression analysis indicated that twig thickness accounted for only a small proportion of the observed variation. This suggests that the assay primarily reflects differences in host response rather than morphological variation among samples.

The protocol described here is largely based on the original excised twig method described by Krzesinska and Azarenko [33] and later used by Li et al. [21], to develop an automated image-based detection system. When adapting the method for peach, several practical differences were observed. Compared to cherry, peach twigs were more difficult to peel due to the thinner outer bark, which increased the risk of damaging the inner bark and exposing the cambium. Rapid oxidation of the exposed inner bark represents another practical limitation, particularly in peach. Following removal of the outer bark, discoloration can occur quickly and may interfere with accurate lesion measurement. For this reason, imaging should be performed immediately after peeling. In our experience, processing samples in small groups and involving multiple people helped minimize oxidation-related artifacts. In addition, for experiments involving a larger number of accessions, temporary storage of shoot segments at 4 °C may stop symptom progression and facilitate batch processing. Consistent with the original cherry protocol, lesion evaluation was performed within the upper 3 cm of the shoot segment. This length was also practical in peach due to the difficulty of peeling longer sections without damaging the inner bark. However, lesion progression may extend beyond this region, and restricting evaluation to 3 cm could potentially reduce resolution among genotypes with more gradual symptom development. As with other detached twig assays, high humidity during incubation may promote secondary fungal growth. When fungal contamination was observed, brief submersion of twigs in a fungicide solution (3 s) effectively reduced surface fungal development without interfering with symptom assessment. This step may be necessary in experiments conducted under prolonged incubation or elevated humidity conditions.

Although detached leaf and shoot assays have been applied in sweet cherry and other *Prunus* species to assess bacterial canker responses [20,21,22,23,24,33], detailed methodological descriptions are often dispersed across multiple publications or adapted for different experimental objectives. By consolidating and standardizing this approach for peach, the present study provides a clearly described reproducible protocol that addresses species-specific considerations and supports breeding-scale germplasm evaluation.

## 6. Conclusions and Future Perspective

This study shows that the adapted detached dormant twig assay can distinguish differences in bacterial canker severity among peach accessions under controlled laboratory conditions. Consistent results across independent replications confirm that the method is reproducible. The protocol described here provides a practical tool for screening peach germplasm and generating phenotypic data suitable for future genetic studies. Future work should determine whether responses observed in the laboratory correspond to actual disease performance in the field. Integration of phenotypic data with genomic or transcriptomic analyses may help identify candidate genes involved in bacterial canker tolerance. In addition, the potential influence of rootstock–scion interactions should be evaluated under field conditions.

## Figures and Tables

**Table 2:** Disease severity observed in the inner bark of detached dormant twigs from nine peach accessions inoculated with *Pseudomonas syringae* pv. *syringae (Pss).* Visual (V) and ImageJ-based (J) measurements are shown for two replicates (R). Data presents ten observations per accession per replicate.

Accession	Visual Score		ImageJ Measurement		Diameter (mm)	
	R1	R2	R1	R2	R1	R2
P1	1.1	1.7	0.093	0.250	0.536	0.449
P2	1.3	1.1	0.100	0.083	0.489	0.417
P3	1.7	2.11	0.164	0.326	0.592	0.479
P4	1.9	2	0.289	0.260	0.543	0.416
P5	2.6	2.9	0.400	0.471	0.53	0.415
P6	2.3	2.2	0.363	0.315	0.51	0.487
P7	2.3	3.7	0.339	0.574	0.501	0.482
P8	3.4	2.2	0.569	0.367	0.393	0.388
P9	4.3	5	0.744	0.857	0.383	0.391
Average	2.32	2.55	0.340	0.389	0.497	0.436

## Data Availability

The original contributions presented in this study are included in the article/Supplementary Materials. Further inquiries can be directed to the corresponding author.

## Supplementary Materials

The following supporting information can be downloaded at: https://www.mdpi.com/article/10.3390/mps9020034/s1.

## Abbreviations

The following abbreviations are used in this manuscript:

PTSL	Peach tree short life
MgCl_2_	Magnesium chloride
*Pss*	*Pseudomonas syringae* pv. *syringae*
ImageJ	Java-based Image Processing Program
CFU	Colony-forming unit
LB	Luria–Bertani medium
OD_600_	Optical density at 600 nm
DI water	Deionized water
Rpm	Revolutions per minute
cm	Centimeters
h	Hours
Min	Minutes
H_2_O	Water
UV	Ultraviolet
MAS	Marker-assisted selection
KMB	King’s B Media
mL	Milliliters
mM	Millimolar
µL	Microliters
C	Control
T	Treatment
°C	Degrees Celsius
USDA	United States Department of Agriculture
AMS	Agricultural Marketing Service
