# Supplementary material for: Detached Twig Assay to Evaluate Bacterial Canker on Peaches
Source: Methods Protoc. 2026 Feb 28;9(2):34. doi: 10.3390/mps9020034 (PMC13010660; doi:10.3390/mps9020034)
Supplement: Supplementary file 1 [file mps-09-00034-s001.zip › mps-4125210-supplementary.pdf]

Detached Twig Assay for Bacterial Canker Response Evaluation in Peach

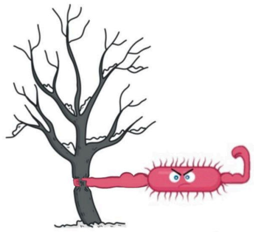

RESERVED DOI:
10.17504/protocols.io.3byl46n62go5/v1

Bilgehan Ahmet Geylani[1], Stephen Parris[1], Jhulia Gelain[1], Guido Schnabel[1], Ksenija Gasic[1]
[1]Department of Plant and Environmental Science, Clemson University, Clemson, SC 29634-0310

Bilgehan Ahmet Geylani
CLEMSON UNIVERSITY

Protocol Info: Bilgehan Ahmet Geylani, Stephen Parris, Jhulia Gelain, Guido Schnabel, Ksenija Gasic . Detached Twig Assay for Bacterial Canker Response Evaluation in Peach. **protocols.io** **https://protocols.io/view/detached-twig-assay-for-bacterial-canker-response-hfu9b3nz7**

Created: November 20, 2025

Last Modified: December 05, 2025

Protocol Integer ID: 233089

Keywords: Pseudomonas syringae pv. syringae, lab assay, ImageJ, phenotyping, disease response, bacterial canker

Funders Acknowledgements:
USDA AMS
Grant ID: AM23SCBPSC1211

Abstract

A detached dormant twig assay, previously applied in cherry, was adapted to evaluate peach germplasm response to *Pseudomonas syringae* pv. *syringae* (Pss). Dormant shoots were cut into 10 cm segments, surface-sterilized, and inoculated with either a *Pss* suspension resuspended in 10mM $MgCl_2$ buffer or with the buffer alone. Following six weeks of incubation, including one week at low temperature to induce ice nucleation, inner bark lesion length was quantified visually and using ImageJ. This assay effectively differentiates genotypes based on lesion development, providing a method to assess tolerance to bacterial canker in peach.

Guidelines

Ensure shoot dormancy is set. At the end of the experiment inner bark will oxidize quickly after outer bark is removed.

Materials

- Gloves (TouchNTuff®)
- 70% alcohol(as a disinfestant) (FisherScientific®)
- Pruning scissors (Corona®)
- Markers (Sharpie®)
- Flagging tape (for bundling each cultivar's dormant shoots after collection, each tape should be labeled with its name on the tape, FisherBrand®)
- Tongs (taking shoots from water and disinfectant(bleach solution), Moacc®)
- Paper towels (KimTech®)
- Plastic plates (for overnight drying, 1 per cultivar) (Sigma-Aldrich®, Disposable polystyrene weighing dishes)
- Plastic pot labels (Hummert International®)
- Parafilm (Parafilm® M Lab Film**)**
- Digital caliper (Mitutoyo®)
- Tube racks (FisherScientific®, Autoclavable test tube racks)
- Floral foam bricks (OASIS Standard Floral Foam Maxlife)
- Plastic containers (for first and third incubation) (Sterilite®)
- Ziplock bags (for second incubation)(SCJohnson®, Ziplock Bags)
- 250 ml beakers (Pyrex®)
- Shaker (VWR®, Advanced digital shaker)
- 1-liter containers (Uline®)
- 4°C refrigerator
- Growth chamber (15°C, 8 hours dark, 16 hours light)
- -2°C refrigerator
- Autoclavable bag (FisherScientific®)
- Autoclavable trays (Nalgene®)
- Scanner(Epson®)
- Bleach (Clorox®)
- Razor blades (for peeling) (Ihc-Tek®)
- Absorbent surface liners (as a bench protector during the peeling process) (FisherScientific®)

Troubleshooting

Problem

Fungal development

Solution

Submerge shoots in fungicide (e.g., Bravo Weather Stik [chlorothalonil] at 5ml/L) for about 3 seconds and then place them back into their original positions inside the containers.

Safety warnings

All tools in contact with the bacteria (e.g., beakers) should be autoclaved. Plastic equipment that could not be autoclaved (e.g., scanner plates) should be cleaned with alcohol or disposed of in biohazard waste for autoclaving (e.g., gloves).

Before start

Make sure all materials are available, incubation chamber is at the correct temperature and day/night setting, and the cold chamber is available and set at the correct temperature.

Pseudomonas syringae Inoculum Preparation

1 **Media and buffer preparation**

1. Prepare King’s B Media (KMB - recipe below) or Pseudomonas Agar F (BD Difco™, Fisher Scientific, Cat. No. DF0448-17-1) plates.

- Add 20 g proteose peptone, 1.5 g K_2HPO_4, and 1.5 g $MgSO_4{\cdot}7H_2O$ to ~900 mL distilled water.
- Add 10 mL glycerol (measure accurately; viscous).
- Ad 15 mg agar powder (BD Difco™, Fisher Scientific, Cat. No. DF0812-07-1)
- Stir until fully dissolved.
- Bring volume up to 1 L with distilled water.
- Adjust pH to 7.2 if needed (usually not necessary).
- Autoclave at 121 °C for 15 minutes.
- Cool to ~55 °C and pour plates.

2. Prepare Luria-Bertani (LB) broth (BD Difco™, Fisher Scientific, Cat. No. DF0446075); pour 20 mL in each 50 ml Falcon tube.

3. Prepare sterile 10 mM $MgCl_2$ buffer:

- 10 mM $MgCl_2$ = 0.952 grams of anhydrous $MgCl_2$ or 2.03 g of $MgCl_2$ x 6 H2O in 1 liter of DI water; autoclave.

2 **Revival and plating**

1. Streak *Pseudomonas syringae* isolate from preservation in glycerol/Cryo media onto KMB/ *Pseudomonas* agar plates.
2. Incubate plates at 25–30 °C for 24–48h.

3 **Colony purity check**

1. Examine plates under 365 nm UV light.
2. Confirm uniform fluorescence (yellow–green).

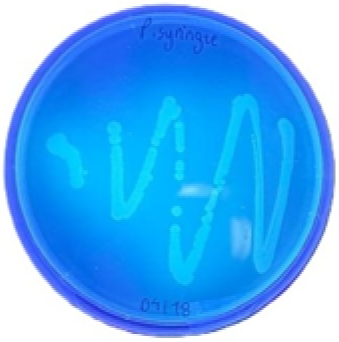

Figure 1. *Pss*-streaked plate under UV light.

4 **Starter culture preparation**

1. Using a sterile loop, pick a single colony from KMB.
2. Inoculate into 20 mL LB broth in sterile culture tubes.
3. Incubate overnight (14–18 h) at 30 °C, 180 rpm.

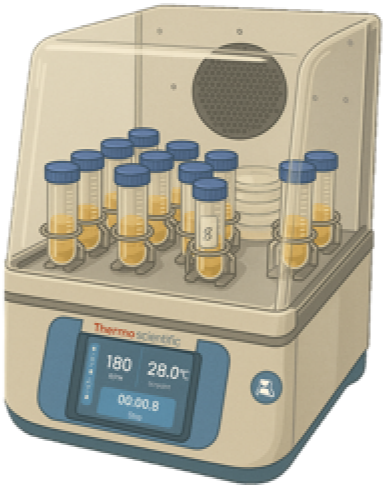

Figure 2. Incubation of *Pss* pre-cultures in a shaking incubator.

5 **Measure starter culture density**

1. Blank spectrophotometer with LB.
2. Measure OD_{600} of the starter culture. Typical values: 0.3–0.6.

6 **Log-phase culture initiation**

1. Transfer 0.2–0.4 mL of the starter culture into 20 mL LB broth.
2. Incubate at 30 °C, 180 rpm for ~6 h to reach early log phase.

7 **Confirm log-phase density**

1. Measure OD_{600} using LB as blank.
2. Proceed when OD_{600} ≈ 0.2–0.3 (≈ 2–3 × 10^8 CFU/mL).

Cell Harvesting and Preparation of Inoculum

8 **Pellet cells**

1. Centrifuge cultures at 5,000 rpm for 10 min.
2. Discard supernatant (LB).

9 **Wash cells**

1. Add 20 mL sterile 10 mM $MgCl_2$ to each pellet.
2. Resuspend by gentle shaking.
3. Centrifuge at 5,000 rpm for 10 min.
4. Discard supernatant.

10 **Final resuspension**

1. Add 20 mL fresh 10 mM $MgCl_2$ to the pellet.
2. Resuspend thoroughly.
3. Transfer entire resuspended volume into a sterile bottle containing 580 mL $MgCl_2$ to obtain 600 mL inoculum.
4. Mix gently. Final concentration ≈ 1×10^7 CFU/mL. Use suspension within 4 hours of preparation.

Verification of Cell Concentration

11 **Serial dilution plating**

1. Prepare serial 10-fold dilutions in 10 mM $MgCl_2$ (10^{-1} to 10^{-7}).
2. Plate 100 µL of 10^{-6} and/or 10^{-7} dilutions onto KMB agar.
3. Incubate at 28–30 °C overnight.

12 **CFU calculation**

1. Count colonies on plates with 30–300 CFUs.
2. CFU/mL = (colonies × dilution factor) / volume plated (mL)

Collection of Shoots (Day 1)

13

1. Collect dormant, one-year-old shoots from trees in the field.
2. Immediately prepare shoots following directions below, or store collected shoots at 4°C Overnight

Preparation of Shoots (Day 2)

14

1. Cut shoots into 20 10cm segments of uniform thickness for each cultivar
2. Disinfest shoot segments by immersing in bleach (0.5% sodium hypochlorite) for 5 minutes, stirring periodically.
3. Rinse shoot segments in distilled water 3x.
4. Set shoot segments on benchtop to dry Overnight .
   - Prevent shoot segment overdrying by loosely covering them with paper towels.
5. Measure diameter of shoot segments at the tip with caliper.

Inoculation of the shoots (Day 3)

15 **Preparation of *Pss* Inoculum**

1. Dispense actively growing *Pss* liquid cultures prepared above in steps 8-10 into 250 ml sterile beakers to a depth of approximately 1.3 cm. Use non-inoculated 10mM $MgCl_2$ solution for the mock inoculation control group (Figure 3).
2. Place beakers onto an orbital shaker (100 RPM) to prevent bacteria from settling.

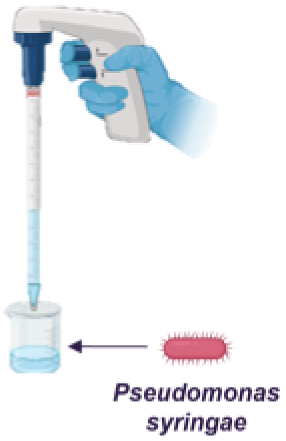

Figure 3. Preparation of *Pss* inoculum by transferring bacterial suspension into beaker.

16 **Preparation of shoot segments for inoculation**

1. Trim 0.5cm from the top of each shoot segment to remove bleach damage and expose fresh tissue (Figure 4).

- Use very sharp pruners or single edge razor blades to ensure clean cut is obtained.

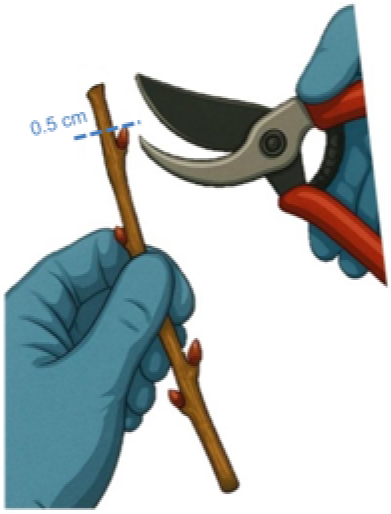

Figure 4. Cutting shoot segments approximately 0.5 cm from the top.

17 For ea**Shoot Inoculation**

- ch cultivar inoculate 10 shoot segments with *Pss* (treatment) and mock-inoculate 10 shoot segments with 10mM $MgCl_2$ solution (control).

1. Submerge the freshly-cut end of prepared shoot segments into the inoculum or 10mM $MgCl_2$ solution for 5 minutes, maintain gentle agitation (100 RPM) to ensure uniform exposure an avoid excessive shaking or frothing (Figure 5).

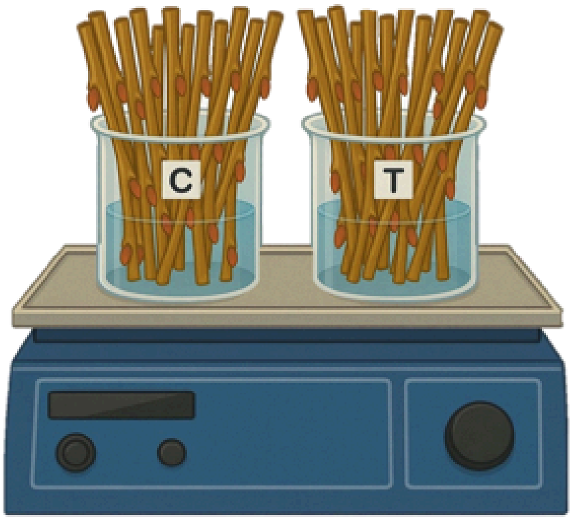

Figure 5. Inoculation setup for shoot segments agitated in bacterial suspension (T) or mock solution (C).

2. Remove shoot segments from beakers and wrap inoculated end with parafilm. (Figure 6.)

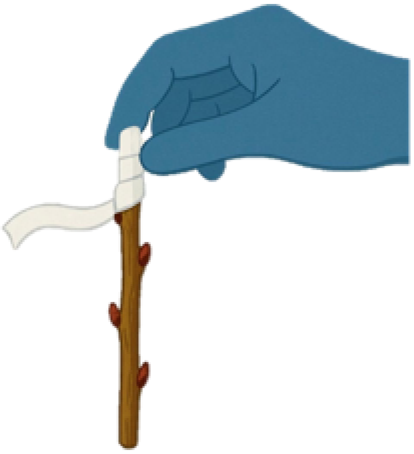

Figure 6. Wrapping the inoculated end of shoot segments with parafilm.

18 **Preparation for Co-cultivation**

1. Label separate plastic containers for treatment and control, place plastic racks in the containers and fill with tap water to 1.5cm depth.
2. Trim 0.5cm from the bottom of the shoot segments to expose fresh tissue.
3. Bundle 10 shoot segments with label by sample and place in plastic racks with freshly cut end submerged in the 1.5cm of tap water (Figure 7).

- Alternatively shoot segments can be separated in the floral foam bricks or individual containers.

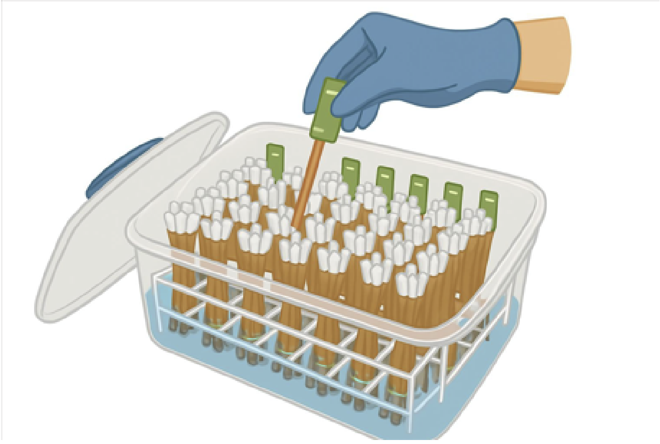

Figure 7. Shoot segments grouped by sample and set in plastic tube racks in a plastic container.

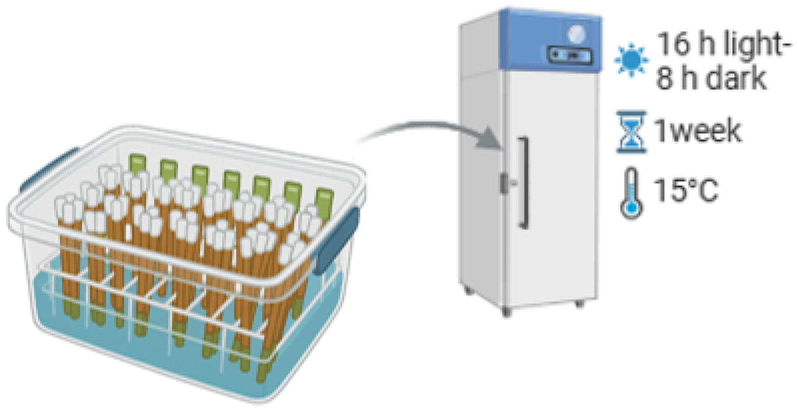

Figure 8. Shoot segments incubation in growth chamber.

Cold Treatment (Day 10)

19 **Cold Treatment**

1. After 1 week of incubation at 15°C, remove the parafilm from the top and dry the base of shoot segments with paper tissue.

- Sterilize the containers with bleach for later use.

2. Place shoot segments into pre-labeled plastic zip-lock bags and transfer to a refrigerator for cold treatment at -2°C in darkness for one week (Figure 9). -2 °C

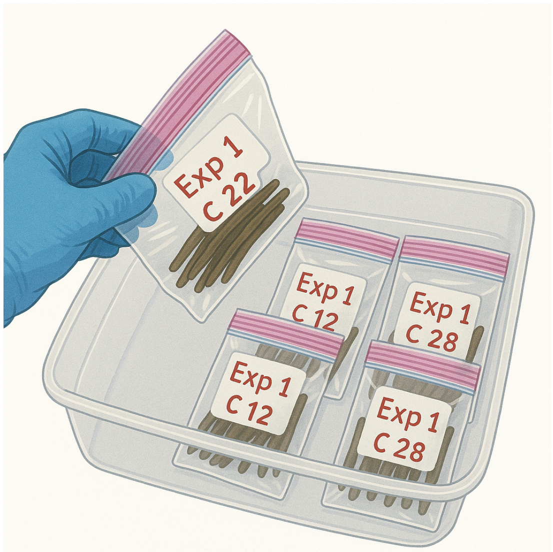

Figure 9. Shoot segments packaged in zip-lock bags for cold treatment (−2°C).

Incubation (Day 17)

20 **Incubation**

1. Prepare sterile containers for incubation by placing floral foam bricks, saturating them with tap water, and maintain 1.5 cm of water level throughout the experiment to ensure adequate moisture.
   - Alternatively shoot segments could be placed in the plastic racks or individual containers.
2. After cold treatment, trim 1 cm from the base of each shoot segment and insert into water-saturated foam bricks up to 2cm of the base.
   - Arrange 10 shoot segments in rows in the foam bricks, and place a label in each row (Figure 10).

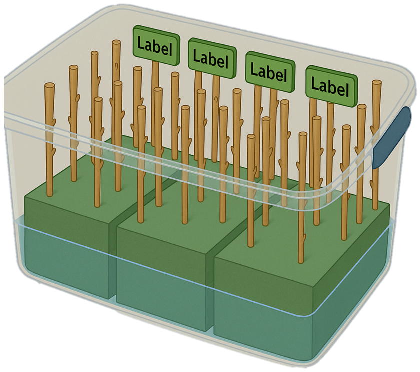

Figure 10. Arrangement of shoot segments in saturated floral foam bricks.

3. Incubate closed containers in a growth chamber for 4 weeks under the same conditions as described under 18.4.

Data Collection (Day 45)

21 **Shoot Preparation and Imaging**

1. Remove shoot segments from experiment and proceed with recording symptom severity.
   - If not evaluating symptom severity at the end of the experiment (too many shoot segments to process) within the same day, place shoot segments in zip-lock bags and store at 4°C to suppress bacterial growth.
2. First, collect an image of shoot segments for each sample using a high-resolution scanner or camera.
   - Arrange the shoot segments on the scanner tray with control at the top and the treatment at the bottom of the tray with inoculated end oriented in the same direction.
   - Scan the shoot sections from both sides.
3. Second, expose inner bark tissue to evaluate the symptom severity.
   - Remove the outer bark with surgical scalpel or single edge razor blade from the top 3 cm of each shoot segment (Figure 11).

4. Trim and collect the top 3 cm of each shoot segment and discard the unpeeled base.

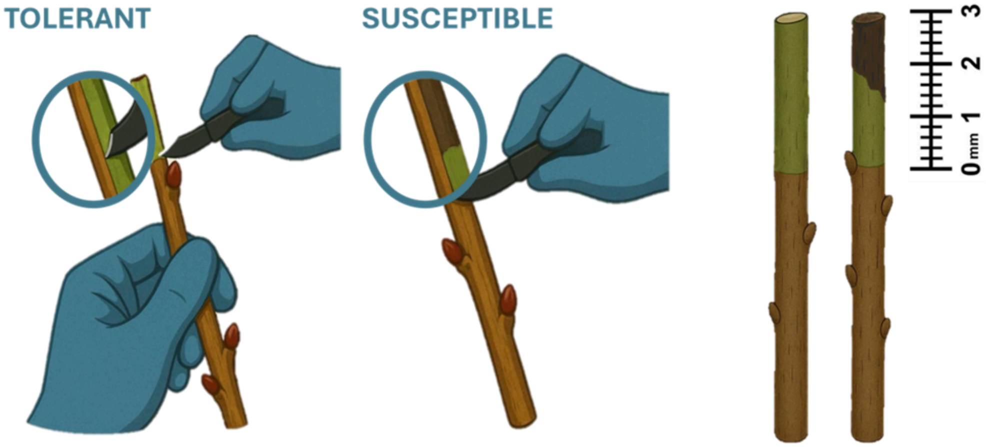

Figure 11. Peeling the outer bark from the top 3 cm of shoot segments.

5. Place peeled shoot segments onto the scanner tray, as described in step 21.2, and collect images (Figure 12).

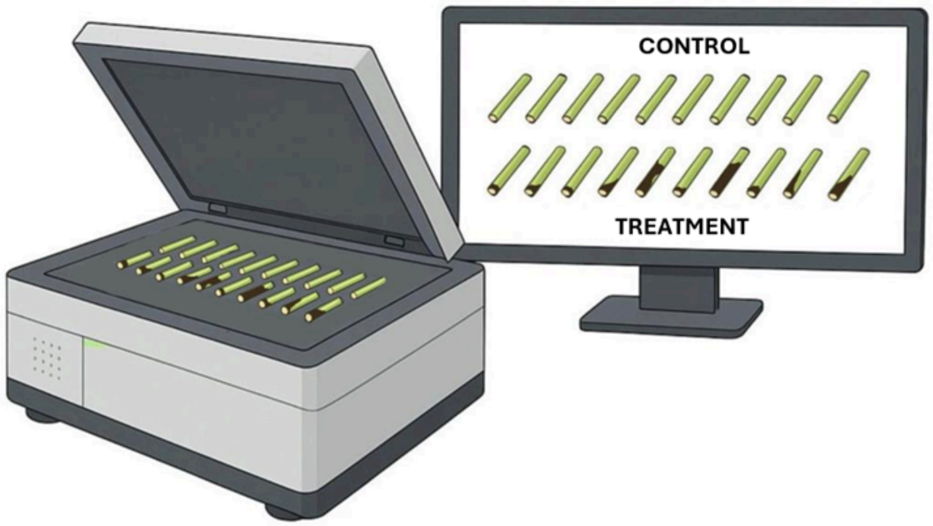

Figure 12. Scanning shoot segments.

Evaluation of symptom severity

22 1. **Visually** evaluate symptom severity, using photographed shoot segments, on a scale from 1 to 5: where 1 represents a sample with no necrotic lesion on inner bark, and 5 represents a sample with inner bark completely covered with a necrotic lesion (Figure 13).

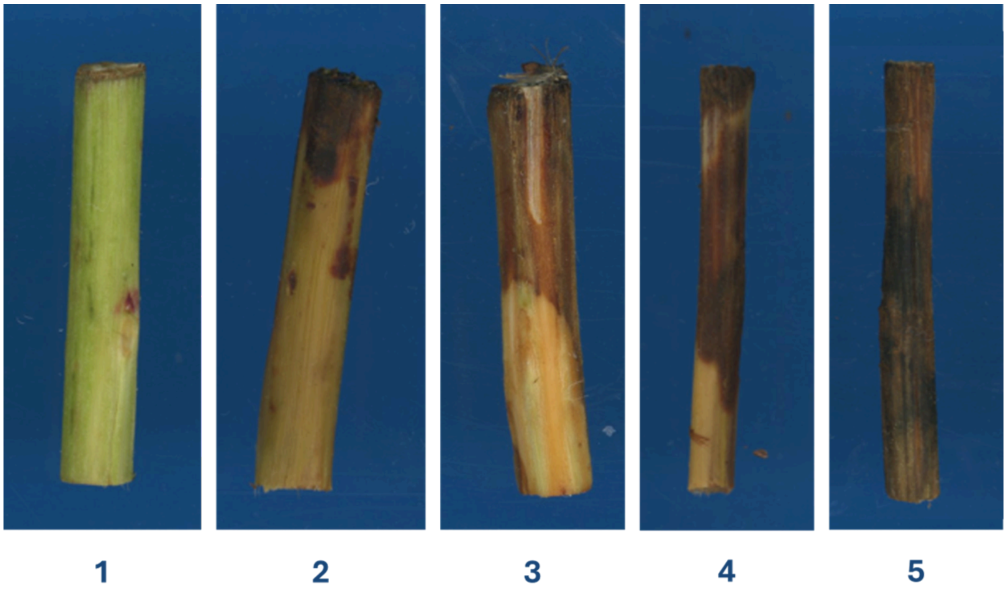

Figure 13. Visual scale for manual evaluation of symptom severity.

2. **Quantitatively** evaluate symptom severity, using photographed shoot segments, by measuring the percentage of each shoot segments' surface area covered by necrotic lesion with ImageJ software (Figure 14).

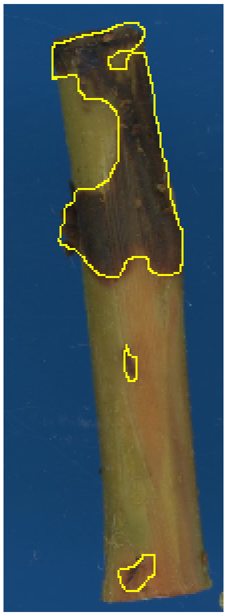

Figure 14. Lesion area quantification using ImageJ software.

Software

ImageJ/Fiji	NAME
Windows 7	OS
National Institutes of Health	DEVELOPER
http://wsr.imagej.net/distros/win/ij152-win-java8.zip	REPOSITORY
https://imagej.nih.gov/ij/	SOURCE LINK

Protocol references

Krzesinska, E. Z., & Azarenko, A. N. M. (1992). Excised twig assay to evaluate cherry rootstocks for tolerance to *Pseudomonas syringae* pv. *syringae*. *HortScience*, **27**(2), 153–155.

Santi, F., Russell, K., Ménard, M. and Dufour, J. (2004), Screening wild cherry (*Prunus avium*) for resistance to bacterial canker by laboratory and field tests. Forest Pathology, 34: 349-362. https://doi.org/10.1111/j.1439-0329.2004.00376.x

Li B, Hulin MT, Brain P, Mansfield JW, Jackson RW, Harrison RJ. Rapid, automated detection of stem canker symptoms in woody perennials using artificial neural network analysis. Plant Methods. 2015 Dec 24;11:57. doi: 10.1186/s13007-015-0100-8. PMID: 26705407; PMCID: PMC4690310.

Acknowledgements

We thank members of the Clemson University Musser Fruit Research Center, Schnabel and Gasic labs for their assistance in this work. BAG was supported by the Republic of Turkey Ministry of National Education. Funding for this project was made possible by a grant agreement [AM23SCBPSC1211] from the U.S. Department of Agriculture (USDA) Agricultural Marketing Service. Its contents are solely the responsibility of the authors and do not necessarily represent the official views of the USDA.
